# Omega-3 Polyunsaturated Fatty Acids and Their Bioactive Metabolites in Gastrointestinal Malignancies Related to Unresolved Inflammation. A Review

**DOI:** 10.3389/fphar.2019.00852

**Published:** 2019-08-02

**Authors:** Pilar Irún, Angel Lanas, Elena Piazuelo

**Affiliations:** ^1^Centro de Investigación Biomédica en Red de Enfermedades Hepáticas y Digestivas (CIBEREHD), Instituto de Salud Carlos III (ISCIII), Zaragoza, Spain; ^2^Instituto de Investigación Sanitaria Aragón (IIS Aragón), Zaragoza, Spain; ^3^Department of Gastroenterology, Hospital Clínico Universitario Lozano Blesa, Zaragoza, Spain; ^4^Departamento de Medicina, Psiquiatría y Dermatología, Facultad de Medicina, Universidad de Zaragoza, Zaragoza, Spain; ^5^Instituto Aragonés de Ciencias de la Salud, Zaragoza, Spain; ^6^Departamento de Farmacología y Fisiología. Facultad de Medicina, Universidad de Zaragoza, Zaragoza, Spain

**Keywords:** colorectal cancer, gastric cancer, esophageal cancer, ω3-PUFA, SPM, IBD

## Abstract

Chronic inflammation takes part in the pathogenesis of some malignancies of the gastrointestinal tract including colorectal (CRC), gastric, and esophageal cancers. The use of ω3 polyunsaturated fatty acid (ω3-PUFA) supplements for chemoprevention or adjuvant therapy of gastrointestinal cancers is being investigated in recent years. Most evidence has been reported in CRC, although their protective role has also been reported for *Helicobacter pylori*-induced gastric cancer or Barrett’s esophagus-derived adenocarcinoma. Studies based on ω3-PUFA supplementation in animal models of familial adenomatous polyposis (FAP) and CRC revealed positive effects on cancer prevention, reducing the number and size of tumors, down-regulating arachidonic acid-derived eicosanoids, upregulating anti-oxidant enzymes, and reducing lipid peroxidation, whereas contradictory results have been found in induced colitis and colitis-associated cancer. Beneficial effects have also been found in FAP and ulcerative colitis patients. Of special interest is their positive effect as adjuvants on radio- and chemo-sensitivity, specificity, and prevention of treatment complications. Some controversial results obtained in CRC might be justified by different dietary sources, extraction and preparation procedures of ω3-PUFAs, difficulties on filling out food questionnaires, daily dose and type of PUFAs, adenoma subtype, location of CRC, sex differences, and genetic factors. Studies using animal models of inflammatory bowel disease have confirmed that exogenous administration of active metabolites derived from PUFAs called pro-resolving mediators like lipoxin A4, arachidonic acid-derived, resolvins derived from eicosapentaenoic (EPA), docosahexaenoic (DHA), and docosapentaenoic (DPA) acids as well as maresin 1 and protectins DHA- and DPA-derived improve disease and inflammatory outcomes without causing immunosuppression or other side effects.

## Introduction

Colorectal, gastric, and esophageal cancers are among the most commonly diagnosed cancers worldwide, as well as the more frequent causes of cancer death. Nowadays, chronic inflammation, caused by failure of the necessary self-limited acute inflammatory response, which prevents from the complete resolution of the inflammatory process, is accepted as one of the main predisposing factors to cancer (Balkwill et al., 2005; Hanahan and Weinberg, 2011). Although CRC cases are mainly “sporadic,” there are several situations in which increased risk has been reported, including genetic and inflammatory disorders. These disorders include inherited mutations in the *APC* gene in FAP, those related to mismatch DNA repair in Lynch syndrome (Ma et al., 2018), or the presence of inflammatory bowel disease (Saleh and Trinchieri, 2011; Dulai et al., 2016). Other factors contributing to chronic inflammation are bacterial infections, such as *Helicobacter pylori* (*H. pylori*) infection related to gastric cancer, or non-infectious causes of inflammation, such as esophageal reflux, the main driver of Barrett’s esophagus and esophageal adenocarcinoma. In addition, other factors include reduced physical activity, an unbalanced diet like those rich in saturated fats, low fiber, red and processed meat, overweight or obesity, alcohol consumption, or smoking, which have been associated with chronic low-grade inflammation (parainflammation) and increased cancer risk too (Baan et al., 2007; Aune et al., 2011; Park et al., 2011; Perera et al., 2012; Aune et al., 2013; Schlesinger et al., 2017; Vieira et al., 2017; Abar et al., 2018). During the inflammation onset phase, endogenous lipid mediators (LMs) like prostaglandins (PGs) and leukotrienes (LTs) are released from arachidonic acid (AA) acting as go signals for inflammation, increasing vascular permeability that enables polymorphonuclear leukocyte (PMN) infiltration into the damaged tissue, and afterwards, prostaglandins (PGE2 and PGD2) acting as stop signals mark the end of acute inflammation and the beginning of LM-class switching process by transcriptional activation of 15-lipoxygenase (15-LOX) in neutrophils and then producing the first class of endogenous specialized pro-resolving lipid mediator (SPM), AA-derived, called lipoxins (LXs), stop-and-go signals for inflammation and resolution phases (Qiu et al., 2001; Nathan, 2002; Serhan, 2007). After LXs, other types of endogenous SPMs derived from ω3 polyunsaturated fatty acids (ω3-PUFAs) presenting as LXs, both anti-inflammatory and pro-resolving properties (Takano et al., 1997; Devchand et al., 2005; Serhan, 2007) named resolvins (Rvs), protectins (PDs), and maresins (MaRs), are produced through transcellular routes by LOX activity, orchestrating the resolution of inflammation during an active process including sequestration of pro-inflammatory cytokines, clearance of neutrophils, phagocytosis of apoptotic neutrophils, and removal of inflammatory debris and restoring tissue (Serhan et al., 2007). Classical anti-inflammatory aspirin treatment, apart from inhibiting PG biosynthesis, can also generate epimeric-aspirin-triggered LXs or Rvs from PUFAs (ATL/AT-Rv) with the same protective actions and longer bioactivities (Gewirtz et al., 2002; Serhan and Chiang, 2008; Serhan, 2014). SPMs exert potent local bioactions and afterwards are rapidly inactivated, presenting short half-lives. For this reason, the elucidation of their chemical structures has provided a model to be used for designing mimetics analogs with reinforced stability, effectiveness, half-life, and an appropriate bioavailability, to be used as pharmacologic molecules to rescue resolution in inflammatory diseases (Serhan and Chiang, 2008). Cancer prevention programs have already been implemented in most countries, but chemoprevention agents should be considered to be used alone or in combination with other treatments to improve resolution of inflammation and prevent cancer development, since once the cancer is present, actual treatments are associated with serious adverse effects and are not effective enough in advanced tumors.

## SPMs in the Resolution of Inflammatory Bowel Disease. Lesson Learned from IBD Animal Models

Inflammatory bowel disease (IBD) is a chronic disease of the gastrointestinal tract presenting two major forms, ulcerative colitis (UC) and Crohn’s disease (CD). UC is a relapsing non-transmural inflammatory condition that affects only the colon (Baumgart and Sandborn, 2007), whereas CD runs with relapsing transmural injuries in several parts of the gastrointestinal tract from the mouth to the anus mainly due to a dysregulated immune response to host intestinal microbiota (Wallace et al., 2014). These disorders are associated with epithelial damage, leukocyte infiltration into the intestinal wall, and AA-cascade activation, increasing CRC risk. Increased risk has been described for bigger extension of inflammation, earlier onset, and longer time from diagnosis (Ekbom et al., 1990; Gillen et al., 1994; Munkholm, 2003; Friedman et al., 2008; Lutgens et al., 2015).The most frequently used IBD models are those generated by induction with 2,4,6-trinitrobenzenesulphonic acid (TNBS) and dextran sodium sulfate (DSS) to resemble CD and UC, respectively (Morris et al., 1989; Bento et al., 2012).

Endogenous lipoxins, the only AA-derived SPMs (Claria and Serhan, 1995), are generated by LOX activity and act as antagonists of pro-inflammatory LTs. Oral administration of ATL analogs reduced weight loss and mortality in DSS and TNBS models and decreased colon injury, colon wall thickening, mucosal PMN infiltration, and mRNA and/or protein expression of pro-inflammatory mediators such as inducible nitric oxide synthase (iNOS), COX-2, macrophage inflammatory protein 2 (MIP-2), tumor necrosis factor-alpha (TNFα), interleukin-2 (IL-2), and IFNγ in TNBS model (Gewirtz et al., 2002; Fiorucci et al., 2004) (**Table 1**).

**Table 1:** *In vivo* actions of synthetic pro-resolving lipid mediators (SPMs), ATL analogs and omega-3 acids in disease models.

Disease model	Actions	Mediator	References
DSS colitis	Reduces body weight loss Improves survival	15-Epi-16-parafluoro-LXA4 (ATL analog)	Gewirtz et al. (2002)
TNBS colitis	Reduces body weight loss Improves survival Reduces colon injury Reduces mucosal inflammation Reduces PMN infiltration Reduces mRNA levels: iNOS, COX-2, MIP-2 Decreases protein levels: TNFα, IL-2, IFNγ	ZK-192 (ATL analog)	Fiorucci et al. (2004)
TNBS colitis	Reduces body weight loss Improves survival Reduces colon injury Reduces PMN infiltration Reduces mRNA levels: iNOS, COX-2, IL-12 p40, TNFα	Synthetic RvE1	Arita et al. (2005)
DSS colitis	Reduces body weight loss Reduces colon shortening Protects the epithelium and crypt architecture Improves disease activity index Induces colonic ALPI mRNA expression Reduces proinflammatory IL-1β and murine KC (IL-8 human homolog)	Synthetic RvE1	Campbell et al. (2010)
DSS colitis	Reduces body weight loss Reduces colon injury Improves disease activity index Reduces PMN infiltration Reduces NF-κB activity Reduces mRNA expression of TNFα, IL-1β, and IL-6	Synthetic RvE1	Ishida et al. (2010)
DSS colitis	Reduces body weight loss Improves disease activity index Reduces colonic tissue damage Reduces PMN infiltration Reduces colonic protein levels of mediators of inflammatory cell recruitment TNFα, IL-1β, MIP-2, and CXCL1/KC Reduces NF-κB activity and mRNA expression Reduces mRNA expression adhesion molecules VCAM-1, ICAM-1, and LFA-1 Potency AT-RvD1 > 17R-HDHA or RvD2	Synthetic AT-RvD1 17R-HDHA RvD2	Bento et al. (2011)
TNBS colitis	Reduces body weight loss Improves disease activity index Reduces colonic tissue damage Reduces PMN infiltration	Synthetic AT-RvD1 17R-HDHA RvD2	Bento et al. (2011)
DSS colitis	Reduces body weight loss Reduces colon shortening Improves disease activity index Reduces PMN infiltration Reduces colonic tissue damage Reduces NF-kB activity Decreases ICAM-1 mRNA expression Reduces IL-1β, TNFα, IL-6, and IFNγ in the acute colitis Reduces IL-1β, IL-6 in chronic colitis	Synthetic MaR1	Marcon et al. (2013)
TNBS colitis	Reduces body weight loss Improves disease activity index Reduces colonic tissue damage Reduces PMN infiltration	Synthetic MaR1	Marcon et al. (2013)
DSS colitis	Reduces colon shortening Reduces colonic tissue damage Reduces colon wall thickness Reduces pro-inflammatory TNFα, IL-1β, IL-6 Reduces PMN infiltration	PD1n-3 DPA	Gobbetti et al. (2017)
DSS colitis	Reduces colon shortening Reduces colonic tissue damage Reduces partially IL-1β Reduces PMN infiltration	RvD5n-3 DPA	Gobbetti et al. (2017)
DSS colitis	Reduces body weight loss Reduces colonic tissue damage Improves disease activity index Reduces PMN infiltration Potency 17-HDHA < 17-HDPAn-6, 10,17-HDPAn-6	Synthetics: 17-HDPAn-6, 10,17-HDPAn-6, 17-HDHA	Chiu et al. (2012)
APC^Min/+^ FAP model	Reduces weight loss Reduces the number of tumors Reduces the size of tumors Increases tissue switch from AA to EPA Reduces tissue prostaglandin levels of PGE2 and 6- keto-PGF1	EPA ethyl ester	Hansen Petrik et al. (2000)
APC^Min/+^ FAP model	Reduces weight loss Reduces lipid peroxidation High reduction in polyp number Reduces polyp load and size Increases tissue switch from AA to EPA Reduces COX-2 expression Reduces β-catenin nuclear translocation Reduces proliferation Increases apoptosis	EPA free fatty acid	Fini et al. (2010)
NMU-colorectal model	Reduces tumor incidence Increases antioxidative enzyme activities of SOD and GPx Reduces lipid peroxidation	Fish oil	Kenar et al. (2008)
DSS colitis	Increases body weight loss Increases colon shortening Enhances inflammation Exacerbates colitis Decreases of adiponectin expression	Fish oil	Matsunaga et al. (2008)
DSS colitis	Reduces body weight loss Reduces colon shortening Downregulates pro-inflammatory TNFα, COX-2, mPGES, TXAS Upregulates anti-inflammatory PGDS Restores the architecture of the colon epithelium Reduces inflammatory cell infiltration Reduces levels of LPO, protein carbonyl and ROS Increases antioxidant activities of GPx, GST and GR	Fish oil	Sharma et al. (2019)
DSS colitis	Reduces colon shortening Reduces disease severity Reduces tissue levels of pro-inflammatory TNFα, IL-1β, and IL-6 Decreases PMN infiltration Reduces NF-kB activity Decreases expression of COX-2 in colon	EPA monoglyceride	Morin et al. (2016)
DSS colitis Fat-1 mouse	Reduces body weight loss Reduces colon shortening Reduces colon damage Reduces PMN infiltration Produces RvE1, RvD3, NPD1, PD1, 17HDHA and 14-HDHA in colon Reduces NF-kB activity Decreases mRNA level of TNFα, iNOS, IL-1β Increases mRNA level of mucoprotective factors Tollip and TFF3	Endogenous conversion of ω6- into ω3-PUFAs	Hudert et al. (2006)
CAC model Fat-1 mouse	Reduces weight loss Reduces colon shortening Decreases inflammation severity and mucosal thickness Reduces tumor incidence Reduces tumor growth rate Reduces NF-kB activity Increases TGFβ mRNA expression Reduces iNOS mRNA expression	Endogenous conversion of ω6- into ω3-PUFAs	Nowak et al. (2007)
CAC model Fat-1 mouse	Reduces tumor number Increases apoptosis Improves inflammation and ulceration scores Decreases ω6 PUFA-derived eicosanoids (PGE2, PGD2, PGE1 and 12-HETE) Increases ω3 PUFA-derived eicosanoid (PGE3) Decreases CD3+, CD4+ T helper, and macrophage cell numbers in colon	Endogenous conversion of ω6- into ω3-PUFAs	Jia et al. (2008)
CAC model Fat-1 mouse	Reduces tumor size Reduces colon shortening Reduces distal colon tumorogenesis Reduces COX-2 protein expression Represses NF-κB transcriptional activation Reduces mucosal PGE2 levels Preserves tumor suppressive 15-PGDH gene expression Reduces proliferation Reduces β-catenin nuclear translocation Increases apoptosis Increases apoptotic molecules FAS and Bax Reduces expressions of antiapoptotic molecules survivin and Bcl-2	Endogenous conversion of ω6- into ω3-PUFAs	Han et al. (2016b)
CAC model C57BL/6 mouse	Similar ω3 tissue PUFAs content and ratio of ω6/ω3 than in the fat-1 mouse Do not confirm anti-tumorigenic effects expressed above	DHA	Han et al. (2016b)
CAC model C57BL/6J mouse	At carcinogenesis initiation: Reduces cell proliferation Reduces β-catenin nuclear translocation Increases apoptosis At carcinogenesis initiation and promotion: Reduces tumor multiplicity Reduces tumor incidence Reduces tumor size Increases tissue switch from AA to EPA Reduces PGE2 Restores the loss of Notch signaling Increases Lactobacillus in gut microbiota	EPA free fatty acid	Piazzi et al. (2014)
Reflux esophagitis model	Reduces esophageal damage Reduces inflammation Reduces expression of MyD88 Decreases pro-inflammatory cytokine expression IL-1, IL-8, IL-6 Increases SOD expression Reduces LPO	Fish oil	Zhuang et al. (2016)
*H. pylori*-associated gastric cancer Fat-1 mouse	Reduces mucosal thickening Reduces inflammatory cell infiltration Reduces gastric inflammation Reduces inflammatory COX-2, IL-1β Reduces inflammatory IL-6, IL-8, IFNγ Decreases angiogenic growth factors VEGF, PGDF Reduces atrophic gastritis and tumorogenesis Decreases gastric cancer Preserves 15-PGDH expression	Endogenous conversion of ω6- into ω3-PUFAs	Han et al. (2016)

ALPI, alkaline phosphatase; ATL, aspirin-triggered lipoxins; AT-Rv, aspirin-triggered resolving; Bax, Bcl-2 associated X protein; Bcl-2, B-cell lymphoma 2; CAC, colitis-associated cancer; COX-2, cyclooxygenase 2; CXCL1/KC, keratinocyte-derived chemokine; DSS, dextran sodium sulfate; FAP, familial adenomatous polyposis; GPx, glutathione peroxidase; GR, glutathione reductase; GST, glutathione-S transferase; HDHA, hydroxy docosahexaenoic acid; HDPAn-6, hydroxy-docosahexaenoic acid; HETE, hydroxyeicosatetraenoic acid; ICAM-1, intercellular adhesion molecule 1; IFNγ, interferon gamma; IL, interleukin; iNOS, inducible nitric oxide synthase; LFA-1, lymphocyte function associated antigen-1; LPO, lipid peroxidation; LX, lipoxin; MaR, maresin; MIP-2, macrophage inflammatory protein 2; MyD88, myeloid differentiation primary response gene 88; NF-κB, nuclear factor kappa B; NMU, N-methyl-N-nitrosurea; NPD, neuroprotection; PG, prostaglandin; PGDF, platelet-derived growth factor 15-PGDH, 15-hydroxyprostaglandin dehydrogenase; PD, protectin; PMN, polymorphonuclear leukocyte; ROS, reactive oxygen species; Rv, resolving; TNBS, trinitrobenzenesulphonic acid; SOD, superoxide dismutase; TFF3, trefoil factor 3; TGFβ, transforming growth factor beta; TNFα, tumor necrosis factor-α; TX, thromboxane; VCAM1, vascular cell adhesion protein 1; VEGF, vascular endothelial growth factor.

Resolvins are endogenous LMs derived from EPA (RvE) and DHA (RvD). As LXs, synthetic RvE1 protects against IBD induction in animal models improving survival, body weight, histological scores of disease by decreasing PMN infiltration, and gene expression of TNF-α, IL-12, iNOS, and COX-2 in TNBS model (Arita et al., 2005) and by the induction of the intestinal epithelial expression of alkaline phosphatase (ALPI) and decreasing phosphorylation of NF-κB p65 Ser276 and mRNA expression of pro-inflammatory TNF-α, IL-1β, and IL-6 in DSS model (Campbell et al., 2010; Ishida et al., 2010). Synthetic RvD supplementation has shown to improve colitis activity index and reduce body weight loss, colonic damage, PMN infiltration, colonic cytokine levels for TNF-α, IL-1β, MIP-2, CXCL1/KC, and NF-κB phosphorylation, as well as mRNA expression of NF-κB and the adhesion molecules VCAM-1, ICAM-1, and LFA-1 in both models. AT-RvD1 showed greater potency than its precursor 17R-HDHA and RvD2 (Bento et al., 2011) (**Table 1**).

Endogenous MaR1 is also a DHA-derived SPM. Synthetic MaR1 has shown similar effects to resolvins in both mentioned models. The mechanism proposed in DSS model suggests the inhibition of the NF-κB pathway and reduction of PMN transmigration and pro-inflammatory mediators like IL-1β and IL-6 (Marcon et al., 2013) (**Table 1**).

Exogenous administration of synthesized PD1_n-3DPA_ or RvD5_n-3DPA_ reduced inflammation and improved the score of disease in the DSS model too, through a mechanism that implies regulation of neutrophil–endothelial interaction and reduction of granulocyte trafficking. The impact of PD1_n-3DPA_ in pro-inflammatory cytokines (TNF-α, IL-6, and IL-1β) was bigger, and RvD5_n-3DPA_ causes only a partial decrease of IL-1β (Gobbetti et al., 2017). Apart from those mediators, other DPA-derived metabolites like 17-HDPAn-6 and 10,17-HDPAn-6, and although in lower degree 17-HDHA, are also effective in protecting from DSS colitis (Chiu et al., 2012) (**Table 1**).

Previously mentioned results are consistent with the protection from DSS-induced colitis found in a mice model that overexpresses the C. elegans fat-1 gene that transforms endogenous ω6 into ω3-PUFAs, resulting in elevated tissue levels of ω3-PUFAs and increased levels of RvE1, RvD3, and PD1/NPD1 (Hudert et al., 2006) (**Table 1**).

In conclusion, exogenous administration of AT analogs and synthetic SPMs has proved effective in improving disease and inflammatory outcomes in most frequently used IBD animal models. Current IBD therapies, based on decreasing signs and symptoms, do not eliminate the disease, cause frequent side effects, are expensive and inefficient in many patients, and cause immunosuppression, like anti-TNFα drugs. Previous results suggest that exogenous administration of stable SMPs derivates might be an innovative and more secure therapeutic approach to control intestinal inflammation, preventing CRC development.

## Omega-3 PUFA Supplementation and Development of Colorectal Cancer and Related Diseases

The possible beneficial effects of ω3-PUFAs in CRC incidence was firstly suggested in 1997 in West Coast fishermen (Schloss et al., 1997). Two years later, it was pointed out that several of the known risk factors for some cancers, including colon cancer, may be reduced by dietary ω3-PUFAs supplementation and encouraged the implementation of clinical chemoprevention trials (Rose and Connolly, 1999).

**Figure 1:**
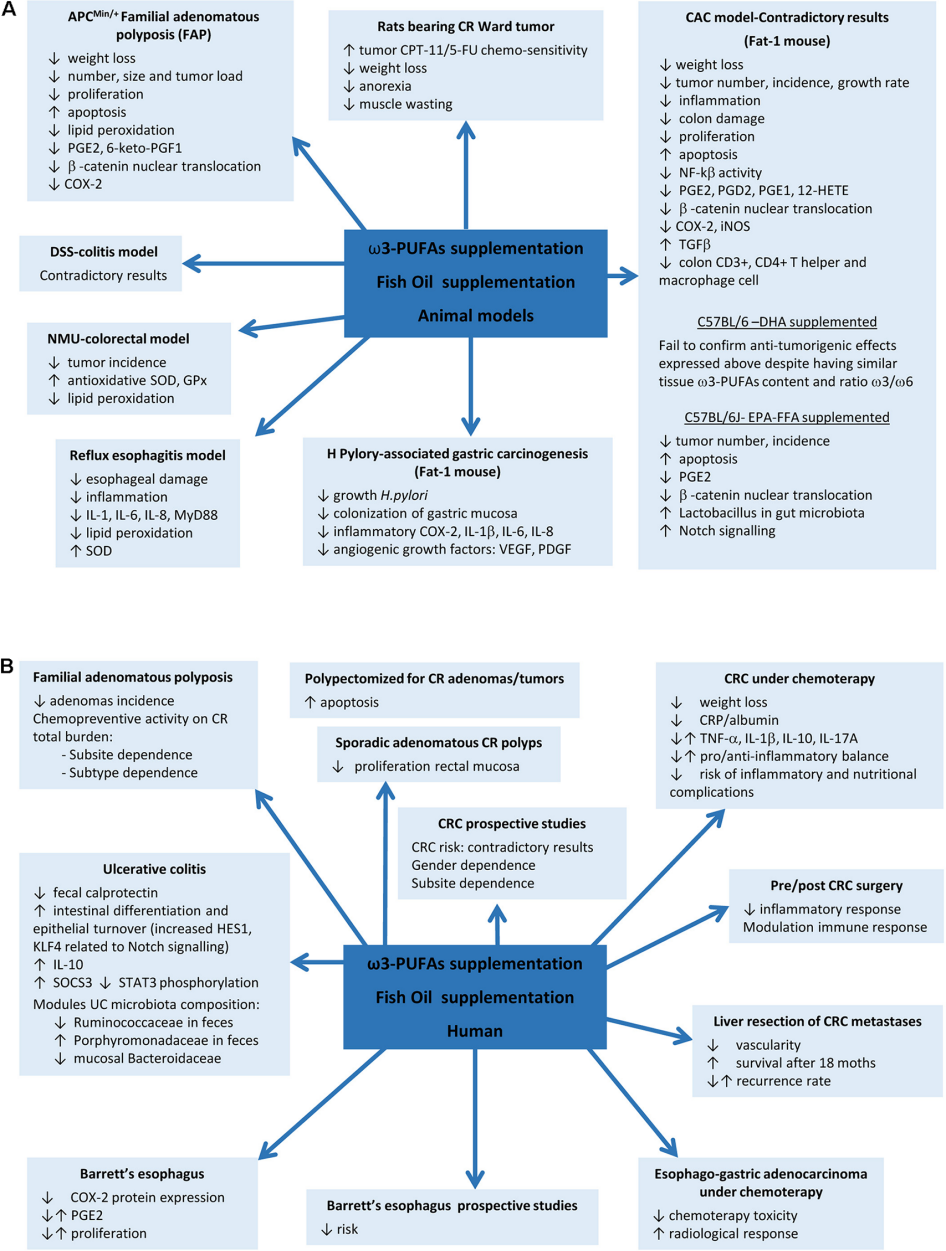
Fish oil or ω3 polyunsaturated fatty acids (ω3-PUFAs) supplementation actions on gastrointestinal diseases. **(A)** Results found in animal models. **(B)** Results in human diseases.

Although a positive effect of ω3-PUFAs supplementation has been reported in some animal models, controversial results have been obtained in DSS and AOM models. EPA supplementation in the APC^Min/+^ mouse model of FAP reported a reduction in the number and size of tumors and improvements on weight, related to COX-2 inhibition, reductions in β-catenin nuclear translocation, and proliferation and increased apoptosis (Hansen Petrik et al., 2000; Fini et al., 2010). Later, protective mechanisms based on upregulation of superoxide dismutase (SOD) and glutathione peroxidase enzymes, reductions on lipid peroxidation (LPO), and downregulated activity of pro-angiogenic genes were also proposed in N-methyl-N-nitrosurea CRC rat model and human colon carcinoma grown in nude mice (Kato et al., 2002; Kenar et al., 2008). However, previous studies in DSS model have yielded contradictory results when supplemented with fish oil rich in ω3-PUFAs or EPA, showing exacerbation of colitis (Matsunaga et al., 2008) or, by contrast, improvement of colitis scores and inflammatory eicosanoids profile, reductions on LPO, ROS levels and PMN infiltration, and increases of antioxidant enzymes (Morin et al., 2016; Sharma et al., 2019). More evidence on contradictory results comes from the mouse model of colitis-associated cancer (CAC) generated by a single pretreatment with azoxymethane (AOM) and posterior ingestion of DSS. AOM/DSS-induced Fat-1 mouse model showed reduced tumor incidence, multiplicity, and size, accompanied by reduction of NF-kB activity, iNOS and COX-2 expression, β-catenin nuclear translocation, overexpression of the anti-proliferative transforming growth factor beta (TGF-β) in colon tissue, reduction of AA-derived eicosanoids, and increased apoptosis, whereas similar ω3-PUFAs content obtained by DHA supplementation in C57BL/6-AOM/DSS model fails to confirm these results (Nowak et al., 2007; Jia et al., 2008; Han et al., 2016b). EPA-protective effects have been also described in non-Fat-1 AOM/DSS model related to restoration of Notch signalling and improvement of Lactobacillus gut microbiota (Piazzi et al., 2014) (**Table 1** and **Figure 1**).

EPA-supplemented long-standing UC patients in stable clinical remission and active inflammation improve endoscopic and histologic scores, intestinal epithelial cell differentiation and turnover, and module gut microbiota composition (Prossomariti et al., 2017), whereas some controversial results have been found between ω3-PUFAs and risk of CRC in prospective studies evaluating fish intake. A meta-analysis of 22 prospective cohorts and 19 case–control studies found in 2012 an overall 12% CRC risk reduction, being more pronounced for rectal cancer (Wu et al., 2012). In 2014, another meta-analysis including 60,627 individuals from prospective and case–control studies showed an opposite association between ω3-PUFAs tissue levels, especially EPA and DHA, and CRC risk (Yang et al., 2014). A study including 68,109 Washington residents found dependence of sex and anatomic subsite, with reduced risk by fish oil supplementation only in men and in colon cancer but not in rectal cancer (Kantor et al., 2014). A later meta-analysis of 14 prospective studies in 2015, including 8,775 patients, found no overall association between ω3-PUFAs intake and CRC risk, in spite of observing a tendency to reduced risk in proximal region and increase in distal location of the colon (Chen et al., 2015). Although controversial results have been found between PUFAs intake and risk of CRC in prospective studies evaluating fish intake, supplementation with fish oil rich in ω3-PUFAs has shown to reduce cell proliferation in rectal mucosa of patients with sporadic CR adenomas (Anti et al., 1992; Anti et al., 1994) and/or to increased mucosal apoptosis (Cheng et al., 2003; Courtney et al., 2007). Probably the best evidence of ω3 supplementation comes from a randomized trial in FAP that found a significant reduction of adenomas incidence (West et al., 2010). The seAFOod Polyp Prevention trial has just concluded that after a year of treatment with EPA and aspirin, the risk of having at least one adenoma is not reduced, but both agents show chemopreventive activity on colorectal adenoma total burden, being EPA more effective in the left colorectum conventional adenomas and aspirin in the right colon, particularly for serrated, but also for conventional, adenomas (Hull et al., 2018) (**Figure 1**).

In relation with surgery, ω3-supplementation during 7 days prior to or after CRC resection reported beneficial effects meanly interfering with inflammatory and immune responses (Liang et al., 2008; Sorensen et al., 2014). Finally, beneficial effects of EPA supplementation have also been found in patients undergoing liver resection for CRC liver metastases, showing reduced vascularity and increased overall survival during the first 18 months after resection, although without changes in recurrence rate (Cockbain et al., 2014) (**Figure 1**).

As colon cancer is particularly resistant to current chemotherapeutic drugs, the role of ω3-PUFAs supplementation as part of an adjuvant therapeutic strategy in colon cancer treatment was soon proposed in order to check their influence in drug toxicity and selectivity. In this way, DHA revealed to be able to selectively target nucleoside analogue arabinosylcytosine (araC) toxicity toward colonic tumor cells without affecting the normal cells *in vitro* (Cha et al., 2005). Similar results were found in rats bearing Ward colon tumor under a cyclical regimen of CPT-11/5-fluorouracil (5-FU) where supplementation with fish oil inhibited tumor growth by raising its chemo-sensitivity and thus decreasing body weight loss, anorexia, and muscle wasting (Xue et al., 2009). Another study has proved the influence of EPA supplementation on the radio-sensitivity of colon adenocarcinoma cells HT-29 by increasing the extent of the LPO caused by radiation (Manda et al., 2011). CRC patients under chemotherapy enrolled in a prospective randomized fish oil supplementation and placebo-controlled study showed reduced CRP/albumin ratio, without changes in inflammatory cytokine profile, suggesting a reduction in the rate of development inflammatory and nutritional complications, and limiting the weight loss, suggesting that supplementation with these compounds is advisable during CRC treatment (Mocellin et al., 2013) (**Figure 1**).

## SPMs in Colorectal Cancer and Related Diseases

SPMs production in the gut is crucial for maintaining homeostasis, and a failure of colonic mucosa to produce adequate anti-inflammatory LMs can explain the persistent colonic inflammation in UC. Colon biopsies have shown important reductions or no detectable production of LXA4 and increased proinflammatory LTB4, PGE2, and TXB2 in IBD patients, probably due to decreased 15-LOX-2 enzyme expression, despite an apparent up-regulation of the resolving and protecting pathways from the ω-3 DPA metabolome. Innovative therapies based on SPMs DPA-derived or aspirin use in order to maintain the capacity to synthesize colonic 15-epi-LXA4 from AA by acetylated COX2/5-LOX have been suggested as good strategies to reduce clinical signs in IBD (Mangino et al., 2006; Gobbetti et al., 2017). A recent report has also found that commercial RvE1 inhibits the oncoprotein c-Myc expression, overexpressed in a large variety of human cancers, and also in CAC model, which causes more tumor aggression and poor clinical outcomes (Nesbit et al., 1999; Beroukhim et al., 2010) in normal human colon epithelial cells stimulated with TNFα and also in HCT116 human colon cells (Zhong et al., 2018). Another recent study has pointed out that chemotherapy generates tumor cell debris, which stimulates tumorigenesis by the release of pro-inflammatory cytokines by macrophages, and that commercial RvE1, RvD1, and RvD2 can turn macrophages from pro-inflammatory/tumorigenic to a phagocytic phenotype, causing clearance of tumor cell debris and then preventing tumor recurrence (Sulciner et al., 2018). In colorectal adenoma recurrence, a randomized trial of aspirin did not found association between plasma levels of LXA4 and RvD1 and the risk of adenoma recurrence despite their previously mentioned anti-inflammatory and pro-resolving actions (Fedirko et al., 2017).

Although a large number of studies correlate the effect of EPA in pro-inflammatory mediator synthesis *via* COX-2 inhibition, it must be said that there is a lack of studies about the situation of SPMs in CRC despite the reported deficiency in one of the enzymes with a strong participation on its production, 15-LOX-1, as the largest contributor to the CRC (Shureiqi et al., 2000; Shureiqi et al., 2005).

## Effect of ω3-PUFAs on Inflammation-Based Cancers of the Upper Gastrointestinal Tract

Gastroesophageal reflux disease (GERD) is a chronic disease caused by the reflux into the esophagus of acid, bile salts, and other noxious agents contained in gastric juice, which induces an inflammatory response and damage of the esophageal epithelium. Complications of reflux esophagitis include the development of ulcers and structures or Barrett’s esophagus (BE), which is defined by the replacement of the normal squamous epithelium by an intestinal type metaplastic epithelium, which is a preneoplastic condition predisposing to esophageal adenocarcinoma (Souza, 2017). The effect of PUFAs has been evaluated in esophagitis, Barrett’s metaplasia, and established adenocarcinoma. Thus, in an experimental model of reflux esophagitis in rats, intraperitoneal administration of a 10% ω3-fish oil-based lipid emulsion significantly decreased esophageal damage and inflammation, whereas administration of a 10% ω6-soybean oil-based lipid emulsion increased the damage (Zhuang et al., 2016). This model is associated with an increased expression of myeloid differentiation primary response gene 88 (MyD88), the proinflammatory cytokines IL-6, IL-8, and IL-1β, and oxidative stress. Interestingly, the authors found the lowest levels of proinflammatory mediators in the ω3-PUFAs-treated animals, whereas the ω6-PUFAs group showed the highest. Both ω3 and ω6-PUFAs reduced the levels of malondialdehyde, a marker of LPO, but the decrease was more pronounced in the ω3-PUFA group, which could be due to an increase in SOD expression, an effect that was exclusive of ω3-PUFAs treatment. A community-based study reported an inverse association between the intake of ω3-fatty acids and the risk of BE, where those who consumed the highest amount were at less than half the risk of developing BE and three times lower the risk to have a long segment BE than those who consumed the lowest amount (Kubo et al., 2009). In a human intervention study, dietary supplementation with 1.5 g/day unesterified EPA for 6 months in patients with BE significantly changed ω3-fatty acid concentrations in Barrett’s mucosa and reduced COX-2 protein expression, although without repercussion on PGE2 levels and cellular proliferation (Mehta et al., 2008). PUFAs also might have a role as adjuvant therapy in established esophageal adenocarcinoma since ω3-PUFAs EPA and DHA have shown anti-proliferative effects on esophageal adenocarcinoma cell lines (Eltweri et al., 2018). A phase II clinical trial in patients with advanced esophago-gastric adenocarcinoma receiving palliative platinum-based chemotherapy showed that the addition of an intravenous infusion of omega ω3-PUFAs as a 10% fish oil lipid emulsion once weekly reduced chemotherapy-related toxicity and improved radiological response (Eltweri et al., 2019) (**Table 1**, **Figure 1**).

In the stomach, *H. pylori* infection is the main risk factor for both gastritis and gastric carcinoma. It is considered to be the initiator of a chronic inflammatory response that contributes to the development of gastric cancer (Park et al., 2015). There is some evidence suggesting a protective effect for ω3-PUFAs against *H. pylori*-associated gastric carcinogenesis. Recent studies have reported that ω3-PUFAs could have antimicrobial activity against *H. pylori*, inhibiting its growth and colonization of gastric mucosa (Correia et al., 2012). Fat-1 transgenic mice overexpress n-3 desaturase, leading to abundant ω3-PUFAS with reduced levels of ω6-fatty acids in their organ and tissues without a dietary ω3 supply. Using a model of gastric tumorigenesis induced by *H. pylori* infection and high salt diet, Han et al. found that Fat-1 mice were protected against *H. pylori*-induced inflammation, chronic atrophic gastritis, and the development of gastric carcinoma compared to wild type mice (Han et al., 2016). Moreover, the expression of inflammatory and angiogenic growth factors such as COX-2, IL-1β, VEGF, and PDGF was significantly decreased in Fat-1 mice. The authors estimated dietary intake of ω3-PUFAs of more than 0.5 g/60 kg to achieve lipid profile similar to that of Fat-1 mice. This study provides relevant preclinical evidence of the effect of ω3-PUFAs on *H. pylori*-induced gastric carcinogenesis and the dose necessary to achieve it (**Table 1**, **Figure 1**).

## Conclusions and Potential Future Developments

Although research on the role of ω3-PUFAs and SPMs on inflammation and cancer is rising continuously and seems to indicate a general positive effect of supplementation on colorectal, esophageal, and gastric cancers, larger efforts should be made to perform high-quality randomized control trials to establish their mechanisms of action, the best timing on supplementation, dosage, source of these products, way of extraction, preparation and quantification, and well-suited nutritional questionnaires to obtain the biggest efficacy, which will allow us to set the use of these compounds in clinical guidelines for cancer prevention.

## Author Contributions

PI revised and summarized bibliography related to colorectal cancer and IBD and contributed to writing the manuscript. AL decided the scope and structure and contributed to writing and revising the manuscript. EP revised and summarized bibliography related to gastric and esophageal cancers and contributed to writing the manuscript.

## Funding

This manuscript was supported by funds from grant PI17/01109 from Instituto Nacional de Salud Carlos III. PI is supported by the CIBERehd. Solutex CG, S.L. has not had any role in funding this manuscript and no role in study design, data collection, analysis, decision to publish, or preparation of the manuscript.

## Conflict of Interest Statement

The authors declare that the research was conducted in the absence of any commercial or financial relationships that could be construed as a potential conflict of interest. Solutex, GC, S.L., a company that produces ω3-PUFAs, contributes to financing the “Catedra de Quimica sostenible” to the University of Zaragoza and research on lipid mediators.
